# The delusive accuracy of global irrigation water withdrawal estimates

**DOI:** 10.1038/s41467-022-30731-8

**Published:** 2022-06-08

**Authors:** Arnald Puy, Razi Sheikholeslami, Hoshin V. Gupta, Jim W. Hall, Bruce Lankford, Samuele Lo Piano, Jonas Meier, Florian Pappenberger, Amilcare Porporato, Giulia Vico, Andrea Saltelli

**Affiliations:** 1 Department of Ecology and Evolutionary Biology, Princeton University, M31 Guyot Hall, Princeton, NJ 08544 USA; 2 Centre for the Study of the Sciences and the Humanities (SVT), University of Bergen, Parkveien 9, PB 7805, 5020 Bergen, Norway; 3 Environmental Change Institute, School of Geography and the Environment, University of Oxford, Oxford, UK; 4 Department of Civil Engineering, Sharif University of Technology, Tehran, Iran; 5 Department of Hydrology and Atmospheric Sciences, University of Arizona, 1133 E. James E. Rogers Way, Tucson, AZ USA; 6 School of International Development, University of East Anglia, Norwich, NR4 7TJ UK; 7 School of the Built Environment, University of Reading, JJ Thompson Building, Whiteknights Campus, Reading, RG6 6AF UK; 8 German Aerospace Center (DLR), German Remote Sensing Data Center (DFD), Muenechner Strasse 20, 82234 Oberpfaffenhofen, Germany; 9 European Centre for Medium-Range Weather Forecasts, Reading, UK; 10 Department of Civil and Environmental Engineering and Princeton High Meadows Environmental Institute, Princeton University, Princeton, NJ 08544 USA; 11 Department of Crop Production Ecology, Swedish University of Agricultural Sciences, Uppsala, SE-75007 Sweden; 12 UPF Barcelona School of Management, Carrer de Balmes 132, 08008 Barcelona, Spain

**Keywords:** Environmental impact, Sustainability

## Abstract

Miscalculating the volumes of water withdrawn for irrigation, the largest consumer of freshwater in the world, jeopardizes sustainable water management. Hydrological models quantify water withdrawals, but their estimates are unduly precise. Model imperfections need to be appreciated to avoid policy misjudgements.

Humans intervene heavily in the global water cycle. The greatest impacts are related to irrigated agriculture, the largest consumer of freshwater resources and a key asset towards food security due to its capacity to maximize crop yields. In the future, irrigation will consume even more water not only to meet the food demands of a growing population, but also to compensate for higher evapotranspiration rates due to climate change.

Quantification of catchment water budgets is thus fundamental to sustainably manage agricultural water resources. Since the late 1980s, when the global scale of water risks became increasingly recognized, policies for sustainable water management have been informed by large-scale hydrological models, which simulate the Earth’s water cycle and quantify the dynamic distribution of terrestrial water resources. The estimates produced by these models have strong policy implications as they feed into the World Water Development Reports, Global Environmental Outlooks and several studies commissioned by the World Bank^1^, in turn shaping local and basin water policies.

Here we argue that the Irrigation Water Withdrawal (IWW) estimates produced by large-scale hydrological models are unreliable. They disregard uncertainties in key parameters and exclude legitimate conceptions of irrigation that do not easily fit within the normative agronomist or engineering mindset, such as that of local and traditional irrigators. This can lead to policy misjudgements and cause social and environmental harm on a vast scale. For example, in 2016, the African Risk Capacity model left ca. six million people in Malawi without water insurance payouts after miscalculating the size of the drought-affected population by more than two orders of magnitude. A later audit revealed that the model used a long-cycle maize variety as reference crop instead of the short-maturing varieties prioritized by Malawian farmers, and that it overlooked the timing between dry spells and the crops’ growth cycle^2^. We contend that a similar disservice can be done to society in its pursuit of the Sustainable Development Goals (SDGs), from Zero Hunger (SDG 2) to Water Stress (SDG 6), if IWW estimates continue to convey an illusion of accuracy.

## The extent of uncertainties in IWW estimates

The estimation of IWW via large-scale models generally follows the relationship between crop yields and water requirement outlined by agronomists and irrigation engineers in the 1950–1960s. In their most simplified form, simulations require data on the extent of irrigation, crop evapotranspiration, precipitation, and irrigation efficiency. Spatially explicit IWW estimates are computed in every grid cell at a given time step and regional, national and global estimates are produced by adding up values at the grid cell level.

None of the relevant parameters can be characterized with precise values. Firstly, the extent of irrigated areas is unknown. There are at least four maps of global irrigated areas based on official statistics and remote sensing imagery, and the values they specify differ significantly^3^. For the same grid cell, the reported irrigated area can extend over 30 ha or over 8000 ha depending on the map used^4^. At the national level, the irrigated area may vary by a factor of two or more. This also applies to top-ranking countries in irrigation water consumption such as China and India, whose irrigated areas range between 43–74 and 15–88 Mha respectively. If we assume an approximately linear relation between irrigated areas and IWW^5^, the IWW for China and India can, respectively, be up to two and six times larger or smaller depending upon the map selected.

**Fig. 1:**
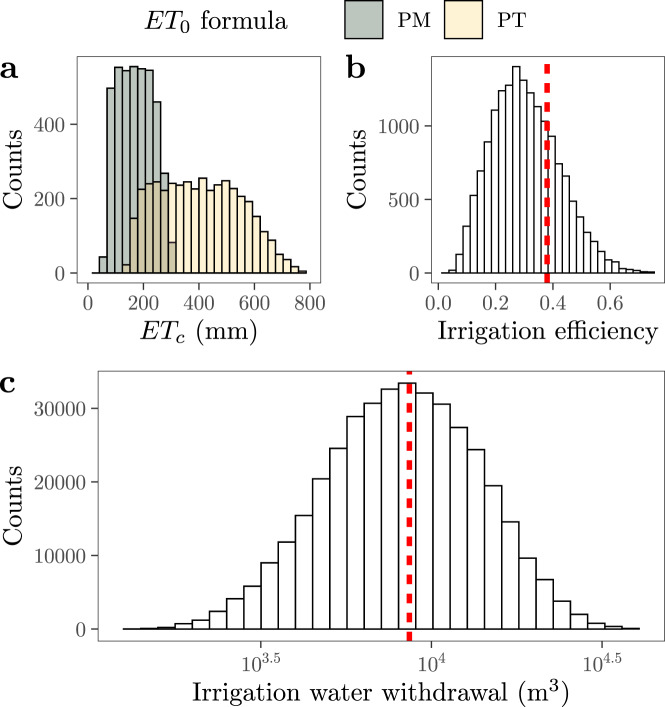
Examples of the ambiguities embedded in the calculation of global irrigation water withdrawals^4^. **a** Uncertainties in the estimation of the crop evapotranspiration (ET_c_). PM and PT stand for the Penman–Monteith and the Priestley–Taylor equation, respectively. Data is retrieved from Nichols et al.^11^. We describe the uncertainty in *k*_c_ with the values reported for salt cedar for May^4^. **b** Distribution of the irrigation efficiency of China after propagating uncertainties. The red, dashed line marks the efficiency value used by some large-scale models^12^. **c** Distribution of the water withdrawn to irrigate wheat in a specific grid cell of the Uvalde County, TX, USA (lon = −99.7083, lat = 29.4583), January 6–7, 2007. All uncertainties in the calculation of IWW are considered^4^. The red, vertical line is the estimate produced when the uncertain parameters used in the calculation of IWW are characterized with point estimates (e.g., mean values).

A similar situation occurs with the crop evapotranspiration ET_c_, which is calculated from the evapotranspiration of a reference crop ET_0_ and a crop-specific coefficient *k*_c_. There are approximately 40 equations available to compute ET_0_^6^ and no agreement as to which one works best in a given context. Since ET_0_ is a modeled variable its value cannot be validated against any measurement at the scale required, which in turn hinders the appraisal of its accuracy even under “perfect” input data^7^. The crop coefficient *k*_c_ is also uncertain as it depends on the crop and its growth stage, but can differ also across individuals and growing conditions. If basic uncertainties in ET_0_ and in *k*_c_ are simultaneously propagated into the estimation of ET_c_, the resulting crop evapotranspiration values may vary considerably. This is shown in Fig. 1a for *Tamarix ramosissima* (salt cedar) in New Mexico (USA), whose potential evapotranspiration in May might be anywhere between 60 and 800 mm.

At a first approximation, crop irrigation water requirements can be estimated as the difference between the crop evapotranspiration and the effective precipitation. The uncertainty inherent in rainfall occurrence and its effectiveness already hinders knowing how much irrigation water may be needed to ensure the development of the crop^8^. And precipitation datasets are prone to ambiguities due to sparsity in the gauge network, the modeling approach, the specific instruments of satellites or the algorithms used to merge data. Their reported annual precipitation estimates over global land can vary by ±100 mm^9^.

Finally, irrigation efficiency is the ratio of the water consumed by the crop to that diverted from the water source to the field. When no water is wasted, irrigation efficiency equals 1. Irrigation engineers assume that efficiencies are mainly defined by the irrigation hardware, with surface, sprinkler and micro-irrigation displaying non-overlapping, increasingly higher irrigation efficiencies. These categories comply with a technology-oriented mindset that disconnects irrigation technologies from their social context while rendering farmers as irrelevant actors in managing irrigation agriculture. Large-scale models sanction this perspective and link countries and/or regions with sharp irrigation efficiency point-estimates that are at odds with the variability of empirically determined efficiencies^10^. Note how the irrigation efficiency of 0.38 used for China by large-scale models turns into a range spanning 0.04–0.77 once basic uncertainties are accounted for^4^ (Fig. 1b). This means that IWW estimates for China can be up to 10 times higher and down to two times lower than current values by simply using an alternative yet equally reasonable irrigation efficiency value.

The simulation of IWW in large-scale models does not consider these uncertainties. Each model typically runs under one irrigated area map, one crop evapotranspiration equation, one precipitation dataset and a fixed irrigation efficiency value per country or region. Parameters are described with point-estimates rather than with probability distributions. This means that the resulting IWW estimates are strongly conditioned by the choices made during the model design and are unreliable.

We illustrate our point in Fig. 1c, where we compare the IWW values produced using the approach of large-scale models (point-estimates) with those produced when uncertainties are propagated with a global uncertainty and sensitivity analysis. We focus on a grid cell in Uvalde (Texas, USA) given the availability of data for all parameters and the strong reliance of the region on irrigation to produce vegetables and grains. We calculate IWW for wheat for just a single day in January^4^. With point-estimates (e.g., mean values) characterizing each parameter, IWW equal 8600 m^3^. With a thorough propagation of uncertainties, IWW estimates range between 1400 and 40,000 m^3^, thus spanning more than one order of magnitude. It is important to stress that this uncertainty affects only one day and one of the several hundred thousand grid cells included in large-scale models. Should this analysis be repeated in all the grid cells and the results added up to compute yearly national and global IWW estimates, the resulting uncertainty ranges in IWW are likely to be of the utmost importance. Simply put, current IWW estimates present us with a mirage of accuracy.

## The way forward

The computation of IWW estimates currently ignores uncertainties. This constitutes a neglect of relevant available evidence and a dangerous complacency in the achievement of the SDGs connected with irrigated agriculture. In our example (Fig. 1c), the use of county-level IWW point-estimates (the red bar) could grossly misdirect irrigation strategies to manage water stress (SDG 6.4.2) because its account of the water required by crops neglects plausible variations and potential extreme values. Omitting uncertainties can also make water managers issue fixed water licenses that do not reflect varying water availability and water requirements’ evolution over time^13^. By the time the error is amended, the social–ecological damage may have become irreversible. We suggest three corrective measures:

First, assume that uncertainties may not disappear. An excess of artificial certainty prematurely locks in a solution, leaving decision makers blind to possible, eventually better, policy alternatives^14^. IWW models are currently pursuing hyperresolution representations (the production of estimates at a 1 km resolution and/or every 1–3 h) to produce more accurate estimates^15^. But this path is unlikely to deliver: greater model detail tends to produce model indeterminacy when uncertainties are thoroughly examined^16^. Increasingly complex models are also likely to usher in more uncertainties due to error propagation and the emergence of new phenomena. This is well known by the climate change community after more than 30 years collecting data on temperature sensitivity to changes in CO_2_ atmospheric concentration, leading to increasing intervals^10, 17^. We argue that uncertainties result from the way we produce knowledge and cannot be banished from science^18^. Rather than trying to eliminate them with more computational power, we might make better progress by developing methodological and conceptual tools to expose them, assimilate them and make them actionable in the real world.

Second, utilize computational power to accurately quantify uncertainties. Uncertainties should no longer be appraised with one-at-a-time (OAT) methods and model ensemble designs^19^. OAT cannot explore the uncertainty space of multidimensional models and completely misses the influence of interactions^20^. Model ensembles do not sample all model formulations and are unsystematic^21^. Rather than sustaining the quest towards ever-realistic models, computational power should be invested in stringent global uncertainty and sensitivity analysis. Apart from Monte-Carlo approaches (Fig. 1c), cost-effective methods such as cheaper-to-run emulators or convergence monitoring allow a much better exploration of the uncertain space than OAT or model ensembles^22^. The outcome will be IWW estimates whose range better matches our knowledge gaps. This is a significant step ahead even if the resulting intervals are too large to be useful for policy-making: in this case, it will suggest that we should leave models aside and turn towards tools better suited to guide policies under irreducible uncertainties, such as deliberative/participative approaches^23^, quantitative storytelling^24^ or robust decision making^25^.

Finally, expose assumptions and value-ladenness. By formalizing irrigation withdrawals as universal equations guided by engineering goals, large-scale irrigation models exclude the values, interests and behaviors of those with the highest stakes in the issue: farmers and irrigators. For instance, the underlying premise that irrigation should optimize crop production and water use is at odds with the goals of traditional irrigators, whose farming practices result in crop diversity rather than maximum productivity^26^. Such differences can amplify the mismatch between what is modeled and the real-world and can eventually lead to a scenario where the model, and not the system of interest, ends up being the object of management. An example is the Chesapeake Bay Program watershed model, whose output was used by regulators to claim that their policies improved the water quality of the main stem of the Bay despite real water monitoring analysis showing no proof of improvement^27^. These biases may be prevented by conducting action-research and involving local knowledge holders in the modeling process. In Pickering, a British town hit by several floods between 1999 and 2007, the participation of stakeholders helped identify upstream storage processes as a critical element initially overlooked by the designers of the flood risk model used to guide decision-making^23^. The result was a model with a higher legitimacy and honed to the social–environmental particularities of Pickering. Local experts with access to national and river basin-scale water accounts may also help assess the accuracy of IWW at the local level, thus enhancing the credibility of subsequent estimates.

Large-scale hydrological models should embrace uncertainties lest they become irrelevant tools for future water management.

## Additional information

Supplementary information, including the code to replicate our results, is available in Puy et al.^4^ and in https://github.com/arnaldpuy/ghm_auditing.
